# Well-Differentiated Thyroid Carcinomas: Management of the Central Lymph Node Compartment and Emerging Biochemical Markers

**DOI:** 10.1155/2011/705305

**Published:** 2011-09-29

**Authors:** Meei J. Yeung, Janice L. Pasieka

**Affiliations:** ^1^Endocrine Surgery Unit, Department of Surgery, Alfred Hospital, Monash University, Brighton, Melbourne, VIC 3186, Australia; ^2^Divisions of General Surgery and Surgical Oncology, Departments of Surgery and Oncology, University of Calgary, Calgary, AB, Canada T2N 2T9

## Abstract

Well-differentiated thyroid cancers (WDTCs) are generally indolent cancers that are associated with a low mortality. Although the incidence of these tumors is increasing, there has not been an associated increase in the mortality rates. As we gain a greater understanding and more experience with these good prognosis cancers, the way in which we treat these tumors is evolving. The definition of persistent or recurrent disease has seen a shift from being a clinical and/or radiological diagnosis to now one based on a biochemical blood marker, thyroglobulin. Central lymph node metastases are a very common problem in WDTC, being present in up to 90% of patients. The optimal surgical management of the central lymph node compartment remains a hotly debated topic. This paper identifies these controversies and presents available data surrounding these issues. Biochemical tumor markers are gaining wider use in practice and in time hopefully provide more specific information with which surgical decision-making can be based. A summary of the clinically available markers is presented.

## 1. Introduction

Well-differentiated thyroid cancers (WDTCs) are the most common endocrine malignancy. The American Cancer Society estimates a total of 44 670 new cases of thyroid cancers to be diagnosed in 2010 and 1 690 deaths related to thyroid cancer [1]. The incidence of thyroid cancer has been steadily increasing over the past three decades [2]. This increase is thought to represent the earlier detection of subclinical disease due to the increased use of office-based ultrasound and ultrasound-guided fine-needle aspiration biopsy. Despite this, the mortality rates for thyroid cancer have remained relatively stable [3]. WDTCs are generally indolent tumors associated with low morbidity and mortality. Although 30-year disease-specific survival rates can exceed 95%, there is a subset of patients with metastatic disease where the 5-year survival can be low as 56% [4]. 

The management of thyroid cancer has evolved over the past decades due to an increased understanding in the pathophysiology of these tumors, analysis of epidemiological data, and the utilization of newer diagnostic tools. With the passage of time, more evidence and thus data, albeit predominately retrospective in nature, is available to assist in directing decision making. The focus now is moving towards minimizing morbidity rather than curing patients of this good prognosis disease.

This paper aims to examine the changes in definition of persistent/recurrent disease with a focus on the controversies surrounding prophylactic central lymph node dissection. In addition, it will look at some of the emerging biochemical markers that present exciting future possibilities in the management of DTC.

## 2. Recurrent Well-Differentiated Thyroid Cancer

The goals of initial therapy of WDTC are to remove the primary tumor, extracapsular disease, and involved lymph nodes [5]. This is ideally achieved with minimal morbidity to the patient. Total thyroidectomy has been shown to result in lower recurrence rates and improved survival for papillary thyroid cancers greater than or equal to 1.0 cm [6]. Utilizing the SEERS database, Bilimoria et al. were the first to demonstrate that tumors greater than or equal to 1.0 cm had a 15% higher risk of recurrence if lobectomy alone was performed. Subsequent studies have supported this data [7, 8]. 

Following surgery, it is helpful to stage patients according to the American Joint Committee on Cancer (AJCC)/International Union Against Cancer (UICC) classification system as this allows for prognostication of individual patients with WDTC and decision making regarding adjuvant treatment, in addition to follow up [5]. The AJCC/UICC classification system stages patients based on tumor size, lymph node involvement, and the presence or absence of distant metastases. This system allows for consistent and accurate communication between health professionals in addition to comparing similar groups of patients in clinical studies. However, thyroid cancers behave differently to other tumors that utilize TNM staging for mortality risk, as seen in the unique age differentiation, where patients less than 45 years of age are never classified as being stage III or above. Most patients with WDTC have an excellent disease-specific survival, and the main concern is related to recurrence, which the TNM staging system does not take into consideration. Consequently, the ATA has developed risk stratification for recurrence [5]. Factors that are associated with tumor recurrence including macroscopic residual tumor, aggressive histology (e.g., tall cell variant, insular cell carcinoma), tumor invasion into locoregional structures, and distant metastasis are taken into consideration [9] (Table 1). Early validation of the ATA recurrence risk stratification recommendations is promising [10].

 There has been a paradigm change in the definition of recurrence from clinically palpable and/or iodine avid disease to a biochemically detectable recurrence [5] (Table 2). Traditionally, disease recurrence was detected on iodine I^131^ scanning [11]. Typically, I^131^ is given approximately 4–6 weeks postsurgery as a diagnostic whole body scan (DxWBS). Better results are achieved when there is little or no residual thyroid tissue, in the presence of thyrotropin (TSH) stimulation and higher doses of iodine [11, 12]. A major advantage of DxWBS is its ability to identify distant metastatic disease.

 Today, serum thyroglobulin (Tg) and high-resolution ultrasound (US) are the modalities used for tumor surveillance [13]. Detectable or rising Tg levels following total thyroidectomy and I^131^ ablation indicate persistent or recurrent disease. Recognizing that thyroid hormone can suppress Tg levels, TSH-stimulated Tg either with thyroid hormonal withdrawal or recombinant TSH (rhTSH) is required for follow-up surveillance. It is important to also measure Tg antibody (TgAB) as the presence of this interferes with Tg measurements by binding to Tg thereby causing an underestimation of serum Tg levels. TgAB is present in up to 25% of patients diagnosed with DTC compared to 10% of the general population [14]. The measurements of TgAB itself may be a surrogate marker for persistent or recurrent disease. In one study that looked at TgAB levels in patients who had undergone remnant ablation with undetectable Tg, 22.6% of these patients showed positive TgAB and 49.0% of these were confirmed with recurrence [15]. 

Head and neck ultrasonography (US) is a first line imaging modality in the investigation of recurrent thyroid disease. The main advantages of US include no ionizing radiation, no known side effects, and easy accessibility. As it does not rely on iodine uptake, it can identify small recurrent disease in the neck regardless of Tg measurements and WBS results. US is usually directed at ascertaining the presence of thyroid bed recurrences and/or cervical lymph node metastases. US is operator-dependent and relies on the experience of the sonographer to differentiate between normal and diseased lymph nodes. Nonetheless, there are features that are associated with pathological lymphadenopathy. These include the size of the lymph node (malignant nodes generally being larger than those that are benign) and the shape (flat is usually benign and round more likely malignant). Other features more commonly associated with malignancy are hypoechoic and the presence of microcalcifications [11]. As with thyroid nodules, when considered in combination, the presence of these features raises concern. The additional use of US-guided fine-needle aspiration biopsy (FNAB) of any suspicious lymph nodes should clarify whether a lymph node is malignant or not. The disadvantages of US include the user dependency and the limited anatomical exposure, for example, the retropharynx, paravertebral, and retrotracheal space. The superiority of recombinant stimulated TSH (rhTSH) Tg combined with US in detecting persisting disease has been demonstrated by Pacini et al. [16]. They were able to show that rhTSH-stimulated Tg alone had a diagnostic sensitivity of 85% for detecting active disease and a negative predictive value (NPV) of 98.2%. When US was added, the sensitivity increased to 96.3% with a NPV of 99.5%. rhTSH-stimulated WBS had a sensitivity of only 21% and a NPV of 89%.

## 3. Management of the Central Lymph Node Compartment

Cervical lymph node metastases are common and are reported to be present in 20–90% of patients with WDTC [8, 17–21]. The risk of regional recurrence is higher in patients with lymph node metastases, especially in those with more than 10 involved nodes and with extracapsular extension [21]. The presence of lymph node metastasis has also been shown to carry a poorer prognosis [22]. 

The cervical lymph node compartments are divided into levels I through VI. These are grouped into central and lateral compartments. The central compartment or Level VI is bounded superiorly by the hyoid bone, laterally by the carotid arteries, and posteriorly by the deep layer of the deep cervical fascia [23]. This level includes lymph nodes from the prelaryngeal, pretracheal, and right and left paratracheal lymph nodes. Surgery for lymph node disease may be considered therapeutic or prophylactic. A therapeutic dissection occurs when cervical lymph nodes are removed after they have been demonstrated to contain metastatic disease based on examination findings, imaging, cytology, or intraoperative assessment. Prophylactic dissection of lymph nodes occurs when there is no evidence of metastatic disease pre- or intraoperatively. Dissection of cervical lymph nodes, whether prophylactic or therapeutic, is performed as a compartment en-bloc resection, with removal of all lymphatic tissue rather than isolated lymphadenectomy (berry picking) as this has been shown to lower rates of persistent and recurrent disease and possibly mortality [5, 24]. 

There is general consensus that clinically involved lymph nodes should be resected, that is, a therapeutic compartment-orientated dissection, to minimize the risk of recurrence [24, 25] and possibly mortality [17]. In contrast, there is considerable controversy concerning the optimal management of the central lymph node compartment [26–29] and whether a prophylactic central lymph node dissection (PCLND) should be performed in patients with PTC. The reason for much of this controversy is related to the lack of large randomized, prospective studies in addressing this issue. The difficulty lays with the altered definition of recurrence in WDTC. Serum Tg levels and high-resolution USs are more likely to detect occult disease. Since lymph node micrometastases are common in PTC, it is conceivable that leaving occult disease in the central compartment would result in a biochemical diagnosis of persistent/recurrent disease. As such, there is a rationale for surgically removing the first drainage basin in a clinically negative neck. Yet, the clinical significance of doing a PCLND has yet to be established. The majority of evidence on which decisions are made are based on nonrandomized, retrospective cohort studies or case series [30]. The information provided in these studies are also disparate in relation to definitions of central lymph node classification, descriptions of therapeutic versus prophylactic dissections, and whether or not postoperative treatment with radioactive iodine (RAI) treatment was given.

Since WDTC is an indolent disease with an excellent prognosis, there is no survival data to support the use of PCLND, as such recurrence rates are the main focus of the current literature. Micrometastases in PTC are common, occurring in up to 90% of examined lymph nodes [31]. Cervical lymph node metastases have been shown to be associated with increased risk of recurrence and morbidity [22, 32]. The preoperative evaluation of the cervical lymph nodes in the central compartment using physical examination alone may miss macroscopic disease in up to 39% of patients [33]. Preoperative assessment with ultrasound is also not able to detect cervical lymph node with a high degree of sensitivity [34]. In a retrospective analysis, CLND was shown to result in lower postablation Tg levels, compared to patients who underwent total thyroidectomy alone [25]. Supporters also contend that reoperation in the central neck for recurrent disease carries greater morbidity with increased recurrent laryngeal nerve (RLN) injury rates and permanent hypoparathyroidism. Yet when CLND is performed by surgeons experienced in this technique, complication rates are comparable to those seen in total thyroidectomy alone. The accepted incidence of permanent RLN injury and permanent hypoparathyroidism is 1% to 2% for total thyroidectomy alone. These same rates are achieved by experienced endocrine surgeons performing total thyroidectomy and CLND [24]. 

The use of postoperative RAI has been recommended to reduce recurrence rates and cause specific mortality [5]. The indication for use of postsurgical RAI for T1 tumors is dependent of lymph node status. PCLND allows lymph nodes to be pathologically assessed. One study was able to demonstrate upstaging from N0 to N1, and the indication for RAI was modified in 30% of patients with T1 tumors [35]. This upstaging has been demonstrated in other studies [18]. In centers that selectively use RAI in low risk patients, PCLND could also potentially decrease the need for RAI in T2 tumors that are proven to be node negative.

Although experienced surgeons have demonstrated similar morbidity rates when performing CLND to that of thyroidectomy alone, surgeons with limited experience perform the majority of operations for thyroid cancer in the United States. As such, the key argument against the routine use of PCLND is exposing the patient to unnecessary morbidity related to the procedure, particularly when evidence exists that does not support a clinical benefit [36–39]. As part of a CLND, the RLN is dissected along its length thereby exposing it to excessive traction and manipulation. Rates of temporary RLN following CLND range from 0 to 25%, with permanent rates ranging from 0 to 11.5% [8]. Permanent hypoparathyroidism exists when vitamin D and/or calcium replacement is required 6 months following surgery. To perform a thorough central lymph node clearance, it is difficult to preserve the inferior parathyroid glands, necessitating autotransplantation. Transient hypoparathyroidism rates with CLND range from 14 to 60% with the permanent rates ranging from 0 to 31% [8, 30]. 

One of the rationales for supporting PCLND relies on the high rate of micrometastases in CLN and potential for increased rated of recurrence if left *in situ* [17]. Yet, the data to date does not support this. Wada et al. [36] studied 259 patients who underwent thyroidectomy and neck node dissection for papillary thyroid microcarcinoma (PMC). Of the 235 patients who underwent a PCLND, 60.9% were found to have microscopic nodal involvement. However only 0.43% of these patients developed a recurrence, with a mean followup of 61.5 months. These patients were compared with a group of 155 patients who underwent thyroidectomy for benign disease and were diagnosed with incidental PMC. This group did not undergo CLND, and they were found to have a recurrence rate of 0.65% with a mean followup of 53 months. In addition, Senyurek et al. [19] reviewed 343 patients with PTC who underwent total thyroidectomy alone. There were 6 (2%) patients with central neck recurrences with a mean follow-up period of 9 years. Interestingly, 3 of these patients had tall cell variant of PTC, 2 had poorly differentiated tumors, and 1 had diffuse sclerosing subtype of PTC.

Although the mortality rates associated with PTC are low, recurrence rates are relatively high. This presents one of the more challenging aspects of managing patients with this disease. The role of PCLND remains controversial, with evidence supporting both sides of the argument. It is important to appreciate that ultimately PTC has a favorable outcome in the vast majority of cases and minimizing harm in these patients is of the utmost priority.

## 4. Molecular Markers

Molecular markers have provided the most promising development in the diagnosis of recurrent and/or metastatic disease. Reverse transcriptase polymerase chain reaction (RT-PCR) is a method that allows the molecular detection of tissue or tumor-specific messenger RNA (mRNA) in the peripheral blood [40]. Numerous genes have been used in thyroid cancer and include Tg, thyroid peroxidase (TPO), thyrotropin or thyroid stimulating hormone (TSH) receptor, and the sodium/iodide symporter (NIS) [40]. 

In 1996, Ditkoff et al. [41] were the first group to use qualitative RT-PCR to detect thyroglobulin mRNA (Tg mRNA) in the peripheral blood. They were able to show a strong correlation between Tg mRNA and the presence of recurrent or metastatic disease. Although this paper provided great hope in the use of Tg mRNA as a molecular marker, subsequent studies were not as successful, with much variability and inconsistency in the results with Tg mRNA [42, 43]. 

Milas et al. recently published their findings following the introduction of the routine use of thyrotropin receptor mRNA (TSHR mRNA) at their institution [44]. Since 2001, their group has studied TSHR mRNA as a molecular marker of thyroid cancer that can be measured from the peripheral blood. In this publication [44], they reviewed 1095 samples from a recent cohort of patients and compared them to conclusions derived from a previous analysis of 663 samples. In their institution, TSHR mRNA is used to guide decision making in patients with an indeterminate FNAB results and has been previously reported [45]. An algorithm based on a combination of the TSHR mRNA value, nodule size, and ultrasound features is used during assessment of an indeterminate lesion. Patients with an indeterminate FNAB result or a follicular neoplasm will undergo a total thyroidectomy as the initial surgical procedure, if they meet one of the following criteria: (i) TSHR mRNA > 1 ng/mcg, (ii) TSHR mRNA undetectable, biopsied thyroid nodule size ≥ 3.5 cm, and presence of one additional unfavorable US nodule characteristic, or (iii) TSHR mRNA undetectable, nodule size < 3.5 cm, and presence of 2 or more additional US features. Unfavorable sonographic features were defined as irregular or indistinct nodule margins, hypervascularity within the nodule, microcalcifications or an abnormal pattern of calcifications, and hypoechogenicity or solid composition of the nodule [44]. By applying this algorithm to model clinical treatment in 54 patients with follicular neoplasms, they were able to show that 28 of 29 (97%) patients with a diagnosis of cancer would have undergone an appropriate initial total thyroidectomy. Milas et al. also evaluated the use of TSHRmRNA as a marker of recurrent disease and in monitoring of goiters. The results are promising but require ongoing surveillance and analysis of data before any clear conclusions can be drawn. Nonetheless, the use of TSHRmRNA may prove to be a useful adjunct in the assessment of patients with thyroid nodules and cancers.

The utility of other molecular marker testing in thyroid cancer has been recently summarized [46]. The BRAFV600E mutation has emerged as an important marker for PTC with high specificity of 99.8% [46]. In their study, Cantara et al. demonstrated that BRAF mutations were always associated with cancer [47]. BRAF mutations have also been associated with extrathyroidal extension, lymph node metastases, increased recurrence, and lower survival rates [48–50]. The future use of BRAF and other forms of genetic testing on cytology specimens may assist in determining which patients require more extensive lymph node dissections, postoperative therapy, and aggressive follow up.

## 5. Conclusion

The assessment, management, and followup of differentiated thyroid cancers continue to evolve as we gain more understanding into its behavior. The definition of recurrence has shifted from the clinical detectable tumor to biochemically detectable disease. When WDTC recurs, it is more commonly detected by a rising serum Tg level or a small focus seen only on US, rather than as a palpable mass in the neck or as uptake on a whole body scan. In the future, more sensitive markers that are not influenced by the presence of TgAB such as an elevated TSHR mRNA level may replace Tg. As our understanding of these molecular markers advances, they may definitively direct our management in determining which follicular neoplasm is cancerous or which malignant PTC will have aggressive behavioral characteristics. These adjuncts may in the future help identify those patients in whom a prophylactic CLND would be beneficial.

## Figures and Tables

**Table 1:** ATA risk stratification for recurrent disease.

Low risk	Intermediate risk	High risk
All the following are present (i) No local or distant metastases (ii) All macroscopic tumor has been resected (iii) No invasion of loco-regional tissues (iv) Tumor does not have aggressive histology (e.g., tall cell, insular, columnar cell carcinoma, Hurthle cell carcinoma, follicular thyroid cancer) (v) No vascular invasion (vi) No I^131^ uptake outside the thyroid bed, if done	Any of the following is present (i) Microscopic invasion into the perithyroidal soft tissues (ii) Cervical lymph node metastases or 31-I uptake outside the thyroid bed on the post-treatment scan done after thyroid remnant ablation (iii) Tumor with aggressive histology or vascular invasion (e.g., tall cell, insular, columnar cell carcinoma, Hurthle cell carcinoma, follicular thyroid cancer)	Any of the following is present (i) Macroscopic tumor invasion (ii) Gross residual tumor (iii) Distant metastases

**Table 2:** ATA definition of disease free status.

(i) No clinical evidence of tumor
(ii) No uptake outside the thyroid bed on I^131^ scans
(iii) No ultrasound evidence of tumor
(iv) Undetectable serum Tg during suppression and TSH stimulation in the absence of TgAB
